# The “Way” Toward E-leadership: Some Evidence From the Field

**DOI:** 10.3389/fpsyg.2020.554253

**Published:** 2020-11-11

**Authors:** Teresina Torre, Daria Sarti

**Affiliations:** ^1^Department of Economics and Business Studies, University of Genoa, Genoa, Italy; ^2^Department of Economics and Management, University of Florence, Florence, Italy

**Keywords:** e-leadership, virtual team, information and communication technologies – ICTs, case study, enterprises

## Abstract

Recently, leadership literature has faced the challenge of dealing with a growing pervasive diffusion of information and communication technologies that are deeply changing relationships among workers. Consequently, leadership is continuing to develop through the support of these technologies. This emerging phenomenon has been labeled e-leadership, and it has been studied with the objective of understanding the differences it exhibits from traditional leadership. Our research seeks to examine whether enterprises, which use leadership as an important “tool” to manage workers as effectively as possible, are conscious of this evolution, whether their behavior is supportive of the related needs, and how they are organizing themselves to face the problems and opportunities arising in this new context. The present study involved 15 Italian companies. Through in-depth interviews based on face-to-face meetings using a semi-structured questionnaire with enterprises’ representatives, we explored the extent of these changes. We developed the analysis across two points in time in order to verify if a change was observable with regard to the way these enterprises considered and managed e-leadership. It was also possible to enhance the role of the technologies themselves in leadership, which in the same period has seen a rapid evolution toward mobile and social developments. Our results help to illuminate that, on the one hand, awareness with regard to e-leadership has increased and, on the other hand, the pervasiveness of technologies is playing a relevant role in the change of leadership together with renewed attention toward soft competencies. We identify four different typologies of e-leadership, which summarize different ways of conceptualizing it, and indicate their main features. We should add that this topic is becoming extremely relevant because of the critical crises organizations are now facing (such as the COVID-19 emergency we are experiencing at the present time) and the urgency of adopting e-instruments, which seem now to be the main path to managing the present situation and the aftermath it inevitably will have. Despite this research being carried out before such an event has happened, we believe that its results may further enrich the current lively debate.

## Introduction

Leadership is a classic topic in the field of management and plays an important role in organizational behavior studies (Bodega, 2002; Knights and Willmott, 2007; Slocum and Hellriegel, 2007). It can be defined briefly as the process by which a person exerts his/her influence over another individual or group to achieve a common goal, with this influence being exercised in an effective way. In fact, effectiveness is the most important condition of successful leadership within this context (House et al., 1999; Northouse, 2007).

In recent decades, the pervasive characteristics of information and communication technologies (ITCs) have changed the way enterprises organize themselves. More specifically, they have permeated the relationship between leaders and followers with an ever-increasing intensity. Therefore, as other authors have suggested (Avolio et al., 2000; Avolio and Kahai, 2003a,b; Dasgupta, 2011; Cortellazzo et al., 2019), leadership is currently developing through the “intermediation” of new ICTs, the presence and usage of which call for a change in the way leadership is practiced. These technologies include the internet, the intranet, e-mail, instant messaging, video conferencing systems, groupware systems, text messages, blogs, document sharing, smart apps, and social media (Avolio et al., 2001; Kissler, 2001; Zaccaro and Bader, 2003; Darics, 2020), all of which are now widespread in almost every working context (Cardon et al., 2019; Roman et al., 2019).

From the academic point of view, even if the call for the study of the relationship between technology and leadership was initiated quite a long time ago (Avolio et al., 2001), the discussion on how leadership has been affected by the digital revolution has so far not been adequately developed, as it would be reasonable to expect (Van Wart et al., 2019). Indeed, while the practice of e-leadership is expanding enormously (Van Wart et al., 2016) and inevitably, either due to the technology itself or the role leadership is expected to play in organizations, the academic Contribution To The Field is still limited (Avolio et al., 2014; Oh and Chua, 2018; Roman et al., 2019). In recent years different constructs such as ‘digital leadership’ have been included in the debate and considered as synonymous for e-leadership (Hüsing et al., 2013; Roman et al., 2019). Whilst the term e-leadership started to become popular from the early 2000s, the term ‘digital leadership’ is a relatively recent one in the domain investigating the relationship between leadership and new technology at work. Indeed, according to our research carried out on Web of Science (WOS), 67 academic works out of the total 89 papers produced in the last twenty years, were published in the period 2016-2020.

More in-depth analysis is required to reduce the gap between the practices in various organizations and the empirical and theoretical studies on such practices (Liu et al., 2018).

Following the current debate (e.g., Cortellazzo et al., 2019; Uhlin and Crevani, 2019), our research contributes to the literature on the relationship between leadership and technology at work. Specifically, it aims at facilitating comprehension of the phenomenon of e-leadership and its definition, starting from how enterprises are aware of the change that is taking place, how they approach it, and how this process is unfolding. Consequently, it points to the importance of understanding if and how organizations are preparing themselves to face problems and opportunities that may arise in this new scenario.

In order to pursue our goal, this paper is organized in the following manner. In the second section, we discuss the theoretical framework on which the research is based. We start from the classic definition of leadership, its main features, and its importance; then, the evolution toward so-called e-leadership is described, focusing on the role played by technologies and the related implications. In this part of the paper, specific attention is devoted to virtual teams, the most appropriate context through which to examine how leadership is exercised, to study how the ongoing change is, at the same time, driven and pulled by technologies, and to determine how people participate (or are pushed to participate) in this process. The third part presents the research design: the research questions are introduced, the method is presented, and the sample is described. Representatives from a group of Italian enterprises, chosen based on their interest in the topic, were interviewed at two different times to assess their experience, analyze the changes observed between the two points in time we examined the topic in our enterprises, and understand their view regarding e-leadership. The following section synthesizes the most relevant aspects of the empirical research. First, we underline the ongoing evolution; then, different situations and behaviors in the selected companies are identified and used to support our proposal of an interpretive scheme of the phenomenon, which can be used to classify different steps toward a full and mature e-leadership.

Finally, some suggestions regarding how e-leadership can be characterized are discussed, limitations and some considerations for helpful future research activities are proposed.

## Theoretical Framework

It has been acknowledged that leadership represents an important phenomenon in social contexts in general and, of course, in organizations in particular. In this section, we summarize its principal features and its development.

### The Evolution of Leadership and Its Relationship With Technologies

In one of the seminal papers on this topic, Avolio et al. (2000) started with the idea that advanced information technologies (AITs)—identified as information technologies with higher levels of basic characteristics and properties of technologies while serving a complementary role to traditional technology—are enabling a new way of working. As research has indicated, leadership is affected by technological change such that the new construct of leadership is more accurately expressed as “leadership in a connected world” (Johnson, 1998), a world where information is disseminated and increases quickly, where time and space are no longer limitations, and where different ways of communicating are continuously developing. In such a context, leadership—which essentially concerns relationships—is transforming. This is occurring first of all because:

• communication uses more and more technological tools;
• information is shared through new technologies, which condition the process of collection, storage, analysis, interpretation, and diffusion of information itself;
• it develops networks that go beyond traditional organizational boundaries, creating new and unexpected relationships.

As a result of these considerations, it has been suggested that “this change requires a significant adaptation on the part of leadership in organizations […], where work is mediated by AITs” (Avolio et al., 2000, p. 615). It has also been underlined that research on leadership must address the new problems arising in the organizational context.

Expanding on this point, Avolio et al. explained that AITs include any tools or techniques capable of promoting multiparty participation through an advanced system of information and knowledge management. Therefore, the authors state (and this is crucial in our perspective) that the effects of technologies are conditioned more by the way they are managed by users rather than by the characteristics of the technology itself (Avolio et al., 2001). The adaptive structuration theory (AST)—which was proposed in the 1990s and grounded on Giddens’ structuration theory—develops a model that emphasizes the social dimension in the interaction between people and AITs. This particular model is based on the fact that a clear idea of the process of organizational development requires the co-evolution of agents and technology (DeSanctis and Poole, 1994; Orlikowski and Iacono, 2001; Jones and Karsten, 2008).

Therefore, while technological change affects the behavior of people, their way of thinking, and their engagement (Wellman et al., 1996), organizational structures, including leadership, which is recognized as one of the most important organizational factors, transforms as a result of the interaction with AITs (Avolio and Kahai, 2003b).

From the individual perspective and according to the unified theory of acceptance and use of technology (UTAUT) (Venkatesh et al., 2003, 2016), the decision to use a new technology by a worker depends on the *intention to use it* that he/she has in organizational contexts. This intention is influenced by four key factors and four moderators (including two individual variables, which are age and gender, and two other personal elements, defined as experience and voluntariness of use). The first of the four factors is *performance expectancy*, which refers to how a person thinks that using a particular technological support will help to improve his/her performance. The second is *effort expectancy*, which is derived from the perception of a person about the complexity or the ease of usage of the technological support. The third is *social influence*, indicating the degree to which an individual perceives the opinions of others as important for his/her decision in adopting the new system—and, evidently, the point of view of the leader has an important effect. The fourth is related to the *facilitating conditions* that are defined as the extent to which a person thinks that the organizational and technical infrastructure can support the use of the system. This perception of support depends in large part on the effort, enacted by organizations, to facilitate the shift toward innovative ways of working, pledging any possible support (e.g., infrastructure and services), and favoring the new leadership “way.”

A leader - who presents herself or himself as an innovator in the use of technology and consequently, creates a positive climate and favorable operational conditions with technology (becoming the advocate to the IT department for any need from one of her/his colleagues about the simple and appropriate usage of available tools) - could easily become an e-leader (Van Wart et al., 2019). In this sense, for example, Neufeld et al. (2007) have demonstrated that charismatic leadership has a positive impact on all organizational factors, which are the facilitating conditions of the UTAUT.

If this is the direction in which organizations have to go, human resource (HR) management also has to evolve. Indeed, it has to follow this change and support the new organizational behaviors arising from it, offering support in understanding how to act within this new context and which competencies are requested (Lengnick-Hall and Moritz, 2003; Bergum, 2009; Parry and Tyson, 2011). It is also crucial to face the potential “disrupting” role of ICT in HR functions, selecting the right set of new technological tools (Sivathanu and Pillai, 2018) and becoming a valuable supporter of the leadership transformation.

### Definition of E-leadership

Recently, the practice of leadership in the virtual realm has become an important part of the daily work of managers. The use of new forms of communication technologies together with the geographical expansion of the activities of firms has increased the need to lead people via digital channels (Darics, 2020). Responding to these changes, organizational scholars have introduced the concept of “e-leadership” to refer to those leaders who conduct many of the processes of leadership largely though electronic channels (Zaccaro and Bader, 2003).

Even if it is a relatively recent phenomenon, e-leadership has become a promising research field of interest and it was a prevailing topic until five years ago. A search for literature on this topic was carried out in the Web of Science (WOS) databank for the past two decades, that is from January 2000 to June 2020. We identified English language published studies including the term “e-leadership” in the title. We found 29 articles, 16 proceeding papers - 8 of which were presented in the last 5 years - 3 editorial material, 2 early access papers, 2 meeting abstracts, and 2 reviews. As we were only interested in original full papers, we excluded literature reviews, comments, abstracts, letters, and editorials. One paper from conference proceedings was excluded because a full journal paper on the same study was obtained in the selection procedure. Eight academic articles, 3 proceedings, and 1 early access paper were excluded from our analysis because their theoretical domain was specifically focused on the educational sector and the relationship between students and teachers in the learning process in educational research. Also 2 proceeding papers and 2 articles were excluded because e-leadership was related to issues not specifically focused on the employee-leader relationship but rather on business strategy, commercialization, and customer attraction. In the end, one proceeding paper was excluded because it dealt with e-leadership applied to e-governance at an institutional level. This search process resulted in 19 articles, 10 proceedings, and 1 early access paper in the field of our interest.

Most of the resulting articles stressed the fact that the topic of the interaction of leaders with followers via ICTs had received limited scholarly attention (see, for example, Roman et al., 2019; Van Wart et al., 2019). Two provided original speculation on the concept and its definition (Avolio et al., 2000; Van Wart et al., 2017) which were also later referred to by works in the field (e.g., Liu et al., 2020). Other scholars further attempted to operationalize the definition of e-leadership considering it as a competence or a set of competencies (e.g., Jones et al., 2017; Roman et al., 2019; Van Wart et al., 2019) also investigating it in specific sectors, such as public administration (e.g., Bergum, 2015) or the navy (Ch et al., 2020).

Quite a good proportion of the studies were also concerned with analyzing e-leadership in relation to the challenge of managing virtual teams (Cascio and Shurygailo, 2002; Zaccaro and Bader, 2003; Chang and Lee, 2013; Fan et al., 2014; Politis, 2014; Ibañez-Cubillas and Miranda Pinto, 2019).

The first and most often cited definition of e-leadership has been proposed by Avolio et al. (2000), who also initiated the debate on the topic. In their work, they state that “past leadership research has not focused on issues confronting the leadership in organizations where work is mediated by AIT” (p. 615) and introduce the expression “e-leadership” with the aim—as they write—“to incorporate the new and emergent context for examining leadership” (p. 617). Following in this direction, they recommend that e-leadership be conceptualized as “a social influence process mediated by AITs to produce a change in attitudes, feelings, thinking, behavior, and performance with individuals, groups, and/or organizations” (p. 619).

Nearly fifteen years after this seminal study, Avolio et al. (2014) indicated that their original definition of e-leadership may benefit from placing greater emphasis on the importance of the context. Accordingly, they proposed a new version, wherein “e-leadership is defined as a social influence process embedded in both proximal and distal contexts mediated by AITs that can produce a change in attitudes, feelings, thinking, behavior, and performance” (Avolio et al., 2014, p. 107). In this second description of the term, they emphasize that technologies operate at two different levels, including: (i) the proximal level, referring to the context which is the closest to the leader and the follower, and (ii) the distal level, which concerns the entire organizational environment and culture.

Therefore, based on this stream of research, leadership appears to be a process of influence mediated by technologies and specifically embedded in the context to which it refers, enlarging its horizon beyond any proximity, which is no longer necessary due to the effects of distance technology.

Liu et al. (2020) summarize the evolution in the definition of e-leadership by focusing on the scope of technology inclusiveness. They start from a “narrow definition” of the scope that is limited to ICTs only, and in which e-leadership is related to the simple use and blending of electronic and traditional methods of communication. The next step refers to the “broad definition” of e-leadership, which includes both ICTs and AITs, and which considers the use of these technologies as a means of support for the organizational processes of knowledge management and decision-making. In the end, in the “grand definition” they provide, Liu et al. further enlarge the scope of the technological inclusiveness by incorporating AITs as evolving organizational structures. Therefore, e-leadership is thereby considered as virtual communication, knowledge management, and the evolution of the system itself because of technology, leading to a “total leadership system” where a continuous interplay and reciprocal influence exists between leadership and technology.

Another complementary view has been offered by Avolio and Kahai (2003a,b), who describe e-leadership as “a fundamental change in the way leaders and followers relate to each other within organizations and between organizations” (2003, p. 50). Their account is focused on the relationships among individuals that continue to be the central element in displays of leadership, but as Avolio and Kahai point out, the expected relationships between people working together are completely transformed by AITs. This particular point is argued coherently along with results emerging from other studies on e-leadership. This change produced by technologies calls for alternative styles from those typical of the so-called traditional leadership, which is facilitated by *de visu* communication based on codes of non-verbal communication and physical presence.

There are some implications of these ideas that have to be considered. The first is the need to develop and manage different communications skills, both in terms of talking and listening. The second is connection to the tools, the level of confidence in their usage, and the capability to finalize the content of the message through the chosen tool. In more detail, the former conditions the latter with respect to the intense usage and development of the tool itself, but the usage of a specific tool also conditions the development of appropriate communication skills, in an evolutionary process of mutual transformation, which reinforces both factors (Avolio et al., 2014).

Van Wart et al. (2019) have more recently proposed a definition of e-leadership which they refer to as a “concrete” and suitable concept for empirical research. Indeed, in doing so, they underline that leaders have to use and blend traditional and innovative tools and styles depending on the different situations that they are in. Furthermore, they have responsibility for the adoption of technologies from their own side as well as that of their colleagues, as suggested by UTAUT with regard to the dimensions of social influence and facilitating conditions. In this line, they suggest that e-leadership is “the effective way and blending of electronic and traditional methods of communication. It implies awareness of current ICTs, selective adoption of new ICTs for oneself, and the organization and technical competence in using those ICTs selected” (Van Wart et al., 2019, p. 83).

Following these previous suggestions, in this paper, we consider e-leadership to be:

• a multidimensional concept characterized by both an individual and an organizational focus and by the capability to concentrate on both the general vision and the details;
• implies, as usual, a social process through which a leader influences a follower (therefore confirming its constitutional intrinsic nature);
• mediated by technologies, the role of which is increasing (and so remarking on the inevitable evolution all of us are experiencing in any working and non-working context);
• a process involving the management of both electronic and traditional methods of communication in an effective and adaptive way (underlying both the coexistence of the two relationships levels and the need for preserving the human dimension), keeping in mind that e-leadership is part of the broader domain of the science and practice of leadership, and that it has to be examined coherently.

As a consequence of the definitions of e-leadership so far considered, effective e-leaders should be individuals who are competent in virtual environments, aware of current ICT tools, capable of choosing them in an appropriate manner, and possess the technical competencies to adopt and use the ICTs selected (Van Wart et al., 2019). They, as well as their own basic communication skills, social skills, team skills, change management skills, and trust building skills, are fundamental to effective e-leadership. In the end, e-leaders should also know how to integrate traditional communication media (e.g., face-to-face communication) with ICTs (e.g., e-mail or videoconferencing). As suggested by Darics (2020), leading people via digital channels requires the combination of various leadership and management functions. Therefore, leaders facing new technologies have to: (1) identify effective working solutions and management processes; and (2) manage people by creating and maintaining the identity of a team by promoting the organizational mission, vision, and values. These two activities are also defined, respectively, as e-management and e-leadership (Roman et al., 2019). This particular conception draws back to the relationship between leadership and management, which are fundamental and related constructs long debated among scholars engaged in these fields (see, for example, Grint, 2005). For the purpose of this work, we consider them to be interrelated insofar as leadership is to be considered as “an integral part of (or embedded in) managerial work” (Larsson and Lundholm, 2010, p. 163), which is to say that managing is part of what a leader does when translating leadership visions into day-to-day operations (Norlyk, 2012).

In this perspective, it seems that the traditional debate on contingent leadership effectiveness, according to which leadership style is based on the following two main orientations highlighted by the least preferred coworker (LPC) scale: a relationship-oriented leader, and a task-oriented leader (Fiedler, 1964), can offer an interesting perspective of analysis. We suggest that the first orientation be better described as *tasks-technology orientation*, while the *socio-relational orientation* maintains its relevance aimed at promoting mutual trust. We maintain that for the “new way toward leadership,” this taxonomy is still effective, albeit with some soft terminological “adjustments” made. Moreover, in order to overcome the challenge of e-leadership, people in organizations are invited to make sense together of the challenges that they face and participate in leadership at every level. It is our opinion the greatest task involved in implementing an effective e-leadership is to create a culture that allows all the voices of leadership to be heard. It should also be emphasized that, according to these considerations, e-leadership appears as a system working at the organizational level and not simply individually performed.

### Virtual Teams and E-leadership

Technological changes have introduced an increase in “virtual” ways of working, as well as in the number of so-called virtual teams in organizations (Hertel et al., 2005; Nydegger and Nydegger, 2010). Indeed, the use of the adjective “virtual” to qualify certain teams emphasizes their strong (and often exclusive) dependence on technology. In other words, web communication and mobile technology—both necessary to reduce physical distance among workers involved in the same team—and individual performance in different places and times are all elements describing the features of a “new” team, in contrast to the traditional workgroup characterized by people “physically” interacting with each other (Avolio et al., 2014).

Zigurs (2002) considers a virtual team to be “a collection of individuals who are geographically and/or organizationally dispersed and who collaborate via communication and information technologies in order to accomplish a specific goal” (p. 343). According to Duarte and Snyder (2006), virtual teams “work without any physical limitation. They normally use collaborative technologies to cut costs and to improve communication and decision timing” (Jones and Karsten, 2008). Accordingly, two necessary conditions for an appropriate definition of a virtual team emerges as follows: (1) the relevance of technologies in facilitating working together; and (2) the distance among the members of the group (Snellman, 2014).

As the role of technologies has already been defined, the question of “distance” calls for a brief analysis. It is indeed considered by many scholars to be one of the basic constituents of virtual teams. For example, Cascio and Shurygailo (2002) proposed a classification of virtual teams based on the location of (one or more) people and on the number of managers involved (one or more), identifying four kinds of work organization, underlining the peculiarity of managing scattered groups, and emphasizing the importance of communication and trust.

Communication is deeply involved in the process of change that the new way of organizing teams produces (Fan et al., 2014). While some scholars point to evidence of the increasing risks of widespread incomprehension among people in the virtual world (Kayworth and Leindner, 2000; Purvanova and Bono, 2009), others analyze the different typologies of communication (depending on whether it is more related to the assigned task or to the relationships among colleagues), thereby concluding that task orientation is essential to reinforcing personal relationships in the new technological context (Hart and McLeod, 2002). This has an important implication, as Brunelle (2013) suggests: organizations have to be careful in the selection of supervisors working in such a context, because—as previously demonstrated (Brunelle, 2009)—specific characteristics are required to enact leadership in the present time. Brunelle points to the capability to balance distance and face-to-face relationships (understanding when it is necessary to privilege direct contact even if more expensive) and the ability to manage individuality and group membership.

Communication is also at the basis of trust. Trust is necessary to manage a team (Lewicki and Bunker, 1996), and, in the case of a virtual team, it is even more important to be mutual among coworkers in order to be effective. It is not by chance that this aspect represents one of the most studied topics in the field. For example, Cordery et al. (2009) have suggested that trust directly influences the performance of a team and invites leaders to take special care of this particular aspect. Moreover, empirical studies have shown that teams with a high level of trust are able to organize themselves better and become productive more quickly (Cascio and Shurygailo, 2002). Therefore, leaders—or, better, e-leaders—have to encourage the creation and strengthening of reciprocal trust (Praveen and Prashant, 2013) through the careful usage of any tools at their disposal (Merriman et al., 2007).

## Empirical Research

### Research Questions

Starting from the above framework, with this study we seek to verify whether and how leadership is evolving toward a new approach with respect to the change in technologies and their usage.

Following our definition of e-leadership, we clarify its distinguishing features and organizational orientation toward a supportive attitude to its diffusion as the new inevitable form that leadership has to assume.

In detail, we seek to answer the following questions:

(i) how do the introduction and diffusion of new technologies influence the leadership system?
(ii) how can leadership foster the usage of new technologies from the perspective of co-evolution and mutual influence?
(iii) which new competencies are necessary for e-leaders?
(iv) what are the roles of the HR and ICT departments in this context?

### Case Study Methodology

The empirical data for this study were collected by applying a multiple case study approach (Cunningham, 1997; Eisenhardt and Graebner, 2007; Yin, 2017). There were two reasons to support the decision to adopt this methodology: (1) the research scope (understanding the evolution of leadership**),** and (2) the research content (the features of this evolution). Regarding the research scope, the case study methodology is consistent with research questions based on “how” and “why” (or the relationship between these two types of questions). Qualitative research is appropriate when the emphasis is on the development of a conceptual framework and the identification of critical factors and other key variables (Eisenhardt, 1989; Mayan, 2016). Thus, regarding the research content, direct contact is essential to understand various elements related to the behavior of people. Multiple cases also allow for the development of a more generalizable and robust theory than a single case (Eisenhardt and Graebner, 2007; Dezi et al., 2018).

The research was conducted according to the guidelines and suggestions for qualitative methodologies provided in the literature (Yin, 2017). Information was gathered through in-depth interviews based on face-to-face meetings using a semi-structured questionnaire submitted in advance to the recipients, which allowed for comparisons across the selected companies, as suggested by Mayan (2016).

The research was carried out during the period of September 2019–January 2020. With the intent to implement a longitudinal approach, we reconsidered and verified the primary and secondary information collected during the first phase of the exploratory research on this topic, which was conducted in 2015 with the same 15 companies. At that time, we met with some (three or four) representatives of each enterprise, who were selected for participation with the help of the HR department among those professionals who were informed about the issues of the analysis, as well as those who were “potential e-leaders.” Each interview lasted 30–40 min to obtain an appropriate level of knowledge of the relevant situations; the contents were recorded, transcribed, analyzed, and then used to develop the idea of an evolving approach toward e-leadership. In the second phase, we met with two representatives, one belonging to the HR department and one to the ICT department, with the interviews lasting for approximately 20 min. When necessary, we carried out follow-up correspondence with the firm’s respondents via e-mail and telephone. In addition to the primary data from the interviews, secondary data from documents (such as business publications, corporate presentations, internet-based information, and newspapers) were gathered. We triangulated these data with the primary data, analyzed the results and their coherence, and reinforced the knowledge of each company. The data were analyzed following the protocols for qualitative data analysis. We guaranteed anonymity to our companies, which were classified with numbers from 1 to 15 for the analysis.

### Case Selection

From the methodological point of view, the research project was organized through a convenience sample of case studies (Eisenhardt, 1989; Yin, 2017). As first, we needed to discuss the topic in-depth with the interested interviewees, so some availability was considered to be necessary as a prerequisite for consideration. For this reason, we prioritized companies with managers attending thematic workshops specifically concerned with new organizational challenges, such as e-leadership, as an index of specific awareness (Conner and Ulrich, 1996; Caldwell, 2003; Nadiv et al., 2017). Then, specific criteria for composing the sample were followed, which included: (i) the sector; (ii) the technological consistency; (iii) the dimensions (according to the European definition of small, medium, and large companies); and (iv) the territorial dispersion (indicated by the number of sites and their location, national or international).

Moreover, we decided to limit our analysis to Liguria, a region in northwest Italy, since its socio-economical context was well known by the authors, and it was a territorially limited context so that companies operating there are subject to similar contingencies. A group of 15 Italian enterprises were involved in our analysis, and Table 1 presents the profiles of the involved companies.

**TABLE 1:** Profiles of involved enterprises.

*Criteria*	*Number of enterprises*
Sector	
Services	10
Manufacturing	5
Technological consistency	
Traditional	6
High tech	9
Dimensions	
Small	2
Medium	4
Large	9
Territorial dispersion	
One site	5
More sites	10
**Total**	**15**

## Main Findings

The principal results are reported below. First, we discuss the situations described by the respondents, with the aim of examining whether debate about and practices of e-leadership were present in our enterprises and to identify evidence of a change between the two points in time. Then, we describe an original proposal to identify some typologies of the e-leadership, which represent different configurations as well as a possible path toward an effective and mature e-leadership. More specifically, four typologies are suggested based on the two leader’s orientations identified in the literature to define leadership styles herein being introduced: the *tasks-technology orientation* and the *socio-relational orientation*. A short description of each typology is offered with regard to suggestions arising from the experiences of our companies.

### Changes in Enterprises Toward E-leadership

Between the two points in time at which e-leadership had been analyzed through interviews with representatives of the organizations involved in the project, some changes were observed. These can be grouped into two main categories.

The first category concerns the *knowledge and pervasiveness of the phenomenon*. When we first approached companies for our research, it was almost always necessary to clarify what we meant by e-leadership so as to help the firms’ representatives focus and organize their ideas and experiences; this is because the concept was a novelty for them. This was true even in cases where the concept was at least known and, in some cases, tentatively practiced (albeit without any minimal formalization) according to the narrative description offered in our interviews. For example, one manager (belonging to company 11) declared, “Of course my leadership is changing … I’m trying to understand how to lead and maintain strong relationships with my co-workers at a distance, using tools and apps we have at our disposal in a creative way. I’m trying, but I’m not sure that I’m doing the best job.” Another respondent (belonging to company 13) asserted, “It is evident that technologies are changing the way we work and interact—I notice that—but I am not aware of what is going on, and I do not see which implications it will have.”

When we approached companies at the more recent point in time, it was evident that there was greater awareness of the construct of e-leadership in many of the companies that had ripened in the interim. Almost all the companies exhibited a good amount of knowledge of the phenomenon, which was also pushed by promotions devoted to the practice as inspired by public events (including conferences and training occasions), and we observed an increased attention to the internal debate about how to implement this “practice” in the management of people. In fact, the majority declared that it would be necessary to prioritize e-leadership as a condition for work in their teams. This was suggested by respondents belonging to companies 9 and 15. With regard to case 15, both the IT manager and the HR manager agreed that there was a need to cooperate in order to help supervisors to reinforce their leadership in the new working conditions. Interviewees of company 15, belonging to the two departments, admitted that there were some difficulties in cooperating one with the other in the same direction, depending on the different cultural approaches they had. However, they were conscious that it was important to help managers to continue playing their role now mediated by technology.

The second category of differences highlighted in the period of examination were related to the dimensions of *technology and organizational culture*. The development of technology, first and foremost with regard to the larger portability of tools and the expansion of connectivity opportunities in response to the ubiquity condition, has fostered a more natural approach to the different communication ways it enables. At the same time, the perception of the presence of leaders has been prioritized, also in distant conditions, who can now be closer to their co-workers. This technological shift is influencing every company, even if those with a limited attitude toward change did not seem to progress as rapidly as they could. In these cases, however, it emerged that the cultural dimension of resistance to change, related to the *status quo* defense, plays a more relevant role than the impressive acceleration observed in technological tools, the diffusion of which belonged more to the personal attitude of a single worker than to the organizational orientation. This situation of “resistance” was evident in companies 1, 3, 5, and 12, thus confirming the importance played by the leaders’ social influence on the attitudes of co-workers toward this process of change, as suggested by the UTAUT. In these companies, respondents reported a lack of confidence in the new way of managing co-workers along with difficulties in using the new tools. For example, the HR manager of company 3 declared, “I think that it is possible to do better using new ICTs, and I try to support who wants to do it… but in my company, the general attitude is not favorable. The dominant idea is that e-leadership is a technological question … Little attention is devoted to understanding if and how tools are really useful. The consequence is that only technology-friendly managers are developing new strategies.”

In contrast to such perspectives, the relationship between e-leadership presence and new technology implementation in companies 8 and 15 seemed to be stronger than it was some years earlier, perhaps due to the fact that they had been fostering a search to find appropriate solutions. This was the shared opinion reported by the ICT and HR managers, who expressed some satisfaction with their cooperation and underlined how they had worked on the culture in their respective enterprises.

### Proposal for a Developmental Path Toward E-leadership

The analysis of the contents of the interviews led to the identification of four typologies of e-leadership. We based these on the two key dimensions of *technology-task orientation* and *relational and managerial orientation*, which we still consider to be a useful way of defining leadership features in the new ongoing context. These types are synthetized in Table 2 and described in the following section.

**TABLE 2:** Our proposal of typologies of e-leadership.

		*Strong technology orientation*	
		
		+	–
*Relational and managerial orientation*	+	E-leadership not present yet	The undeclared e-leadership
	–	E-leadership rooted in the ICT department	E-leadership and virtual team

#### When E-leadership Is Not Yet Present

In five of the analyzed companies, the presence of e-leadership was excluded as reported by the companies’ representatives. These included cases 1, 2, 3, 5, and 12. However, from the first to the second step of the research, it was found that the number of cases that were not oriented to the use of e-leadership had decreased (cases 7 and 13 had adapted to e-leadership).

Even if there was some general awareness that the increasing pressure arising from the wide diffusion of new technologies would lead even these companies, probably in the near future, to reconsider their organizational systems of leadership, it seemed that they were not looking to accelerate the process. In other words, while technologies were recognized as having an important and relevant impact—none of our interviewees denied this fact—they did not yet represent, at least for these five companies, the basis of a new way to manage and inspire co-workers, and were even less likely to be thought of as a new channel through which leadership could be exercised. Such measures were to be implemented “if and when it is strictly necessary,” as the ICT managers of company 3 and 5 reported in unison, indicating that the underlying difficulties in positive implications of some tools were becoming evident.

The preference for a traditional approach that prioritized direct and visual relationships was clear in these companies. This approach hinged upon the centrality of the headquarters, from which everything emanated, and from which people and projects were managed. As reported by the manager of company 5, this perspective was “the consequence of a strong and traditional corporate culture, developed in some cases around manufacturing activities where the leader works together with his/her team in realizing prototypes and organizing their large production, and which does not consider other ways of establishing relationships as concretely or effectively possible.” In other cases, such as that of the services enterprise company 12, the dominant idea was that “working face-to-face facilitates direct and continuous communication. Trust is built up over time and is essentially based on the figure of the leader and his/her recognized professional competencies; co-workers observe the skills of the leader and learn with their eyes.” Innovative tools were increasingly used but without a clear strategic orientation finalized to improve working conditions and activities, so that until that point, they had been classified as “second best.” Workers were requested to report and confront each other in person, as this was the preferred approach. As the HR manager of company 3 indicated, “Nothing is better than seeing people, so as to understand how things are going…”

Thus, it is evident that in these conditions, specific skills for e-leadership were not necessarily encouraged but were rather accepted as unavoidable. Indeed, both the ICT and HR managers of company 5 argued, with different and opposite expectations (positive for the first and negative for the second), that “it is only a question of time…” In this way, we can classify this last typology as “not yet.,” wishing to underline that e-leadership is expected to become a reality to face.

#### When E-leadership Is Rooted in the ICT Department

In some of the other cases we observed—exactly four out of the fifteen analyzed, and precisely cases 4, 6, 10, and 14—the respondents indicated that the concept of e-leadership was well in place and was immediately associated with the ICT department. The introduction of new technologies and their intensive usage represented the basis for the digital transformation in organizations, and served as a major premise in the argument for a more diverse way of changing work organization and implementing teams.

In these organizations, the “first” e-leader was the Chief Digital Officer (CDO), an organizational role in which one works directly in connection with the Chief Executive Officer to express relevant interests and build an appropriate organizational commitment toward this process of change. The CDO was in charge of supporting the diffusion of technologies into the company to enable supervisors to become e-leaders, which means that such an individual must be a technology expert and therefore he/she helps others to use the relevant tools and strengthen their expertise.

Pushed by the CEO toward this organizational development and suggested to become its promoter- the ICT department has to change its approach; traditionally, it has closed in on itself and focused exclusively on the technical perspective, but it is now called on to promote the disruptive change produced by the new digital technologies and to make them interesting for people; individuals within this sphere must support the interests of workers and supervisors. Then, a strong commitment, together with more flexibility and creativity, are necessary to take advantage of these changes and to promote a different way of performing in organizations.

According to this perspective, the e-leader is a person who makes use of ICTs to create relationships, promote communication, and accelerate every process in which he/she is involved. This is the reason why ICT departments have to anticipate this expected situation and recruit young generations of workers into new and unknown ways of managing teams and developing work; this often involves following the diffusions of social media and finalizing them into new ways of sharing in work. Interviewees of companies 4 and 6 described this approach by using the same words, suggesting that “e-leadership means first of all a strong technology orientation,” with the HR manager adding, “… but this has to be useful to improve relationships so as to achieve better common goals.” In the same direction, respondents of company 10 indicated that “our technological mastery—we work in a high-tech sector—has to be evident internally as well; e-leadership begins for us by showing the potentialities of new technologies and documenting how fruitful they are, and these facts are always surprising for line managers… to become leaders is their choice, but building their e-leadership depends on our effort.”

#### The “Undeclared” E-leadership

In three of the fifteen cases analyzed (precisely 7 and 11, which in the first phase displayed some ignorance of the phenomenon, as well as 13), it was evident that e-leadership was carried out, but it had never been formally introduced and was even less officialized by the headquarters. In these enterprises—usually organized with a number of geographically dispersed sites and with relevant problems of coordination among the different activities developed within those sites—the necessity of frequent meetings and *vis à vis* encounters stood in contrast to the concrete possibility of organizing them because of the costs incurred and the time taken away from other productive activities.

Hence, integrating working activities through virtual meetings and cultivating attention, friendship, and confidence in ICTs became a natural way of organizing work. Managers in different areas of the company had reportedly begun to interact with their colleagues spontaneously through technologies, by using virtual conference tools, learning to manage distant co-workers, promoting the habit of new ways of working together, using new supports (including apps and social media), introducing a cultural change in their own attitudes and behaviors as well those of co-workers, maintaining a strong focus on the content of the relationships, and considering technologies as a useful support and not a priority.

In this view, the role of the e-leader stood out naturally due to the managers responding to their own need, to which the ICT and HR departments offered some help. The first department can support in the development of technical aspects, the second can intervene in order to support the improvement of those soft competencies needed in mediated contexts to facilitate positive relationships with colleagues in this process of change. As the interviewee of case 11 declared, “we see that e-leadership is inevitably increasing in relevance in our company…we have always cared about leadership as an overriding dimension of managerial work, and we have struggled in understanding that it is changing, but now we care for it in the new form it is taking.” Also, from company 13, we received a clear message “it has been a surprise for us to see that we practice e-leadership without knowing it… for the future, we think that it is not necessary to formalize it, but it is important to help those who think that it is better in this way.”

#### E-leadership and Virtual Teams

Some enterprises in our analysis, namely companies 8, 9, and 15, were explicitly involved in the direction of establishing an effective and diffused e-leadership. Assertions such as “this is an important issue for our organization” were frequently expressed by the representatives of the cases we include in this typology.

For these companies, e-leadership did not simply entail the mastery of technologies, nor was it considered to be a privileged field for the ICT department, even if a diffused friendship with tools and the suitable ways of using them was a feature of such leadership. As an interviewee belonging to company 9 reported, “it is by now impossible to think that work in our enterprise can be managed without orientation toward e-leadership, and what this means for us is that traditional and innovative paths are followed according to the situation.” Another respondent of company 15 added, “we are interested in supporting the focus on goals, so any way is okay; experiencing new tools so as to guarantee a strong relationship with co-workers has become normal.”

In these contexts, e-leaders are managers engaged in different specific organizational areas; they are required to take on a “new” job, possessing the basic and fundamental features of a traditional leader but also exhibiting a particular level of attention devoted to distance implications and to the changes in relationships resulting from distance. At the same time, he/she has to understand the opportunities and potentialities of technologies, be comfortable with them, and act positively with respect to the perception of usefulness in work and on the confidence of colleagues with technology. Such leaders must also demonstrate an ease of usage of technology while following the suggestions advanced by highly skilled colleagues in the area of technology who introduce emerging tools from their daily experiences. E-leaders must be open to discussing opportunities for their usage given that they can improve work and relationships.

The e-leader is also required to develop new ways of communicating with colleagues by using new tools and shaping communication content in terms of new styles and paces. In other words, using technologies, and specifically collaborative technologies—especially those that are increasingly widespread—makes it easier to work together. The relevance of the role of an e-leader is particularly strong when teams are composed of people living in different places, whereby video conferences, e-mails, and instant messages represent the normal way to communicate. Thus, ensuring engagement and commitment from all the members of the group is the first task for an e-leader, who has to build a positive relational climate and reciprocal trust in conditions where distance can make relationships even more difficult (depending, for example, on differences in time zones, possibilities of misunderstanding, and non-verbal communication, just to mention some barriers). On these requirements, an interviewee belonging to company 15 said “it has been a necessary and great commitment on the part of the entire organization to support this new orientation. It has to become evident that the conditions supporting e-leadership have to be at the organizational level, in other words the organization has to focus on them.”

Within this context, the constructs of “control” and “delegation” change their profile and content; this is because in a distance relationship, everything has to be well defined and clarified, and the preferential way to manage becomes the assignment of goals. Accordingly, another respondent of company 8 underlined that “control is no longer possible as a continuous activity; goals have to be verified, while delegation represents a new and effective task for managers.”

In a certain manner, in these situations, e-leadership is considered unavoidable. For this reason, the conscious choice is to anticipate its evolution and facilitate the transition toward its full realization. Therefore, e-leadership is encouraged in such companies, and it is generally supported by the CEO, so that the role played by managers will ensure that these features are officialized and that the required competencies are developed by training and other practices, thereby foreseeing the consequences that this new way of managing people may have on the business.

### Some Answers to Our Questions

Based on the evidence that emerged from interviews and the resulting description of the four typologies of e-leadership herein reported, answers to the four research questions can now be produced. These answers are summarized in Table 3.

**TABLE 3:** The different features of the four typologies of e-leadership.

	E-leader not present yet	E-leader in the ICT dept.	Undeclared e-leadership	E-leader and virtual team
*How does new technologies (NT) influence the leadership system (LS)?*	No influence.	The introduction of NT is through the ICT who is the “ferryman” of this organizational change and of the construction of a new LS.	The introduction of NT is through the daily practice of managers who are self-identified e-leaders and develop an ‘informal’ LS.	Direct and strong influence, but also a search for support in technologies to develop a more effective leadership.
*How can leadership foster the usage of new technologies in a perspective of co-evolution and mutual influence?*	No mutual influence.	E-leaders have to work as promoters of the usage of technologies as improvement occasions of working conditions.	Through a personal and committed usage of technologies by leaders the mutual influence is reinforced.	Leaders search for support in technologies to develop a more effective leadership.
*Which new competences are necessary for e-leaders?*	Specific skills not encouraged (but accepted as unavoidable).	More flexibility and creativity are requested for IT roles engaged in e-leadership diffusion.	Orientation toward innovation, which means attention to the usage of not-specialized technologies as effective tools.	A clear vision for the role of leadership so to blend traditional skills and innovative ones.
*Which is the role of the HR department and of the ICT department in this context?*	The HR and the ICT dept. are subordinated to a strong and traditional corporate culture based on manufacturing activities.	The ICT dept. has a pivotal role supporting the necessary diffusion of technology and enabling leaders to become e-leaders.	The HR and ICT dept. are supportive departments for the ‘own innovative initiative’ of the managers.	The HR and ICT dept. are supportive of the ‘innovative initiative’ at the organizational level and offer a formal framework to manage it.

Very briefly, we can observe that new technologies, which are increasingly used to make leadership more effective, play a relevant role in influencing it and acting as a facilitator, also increasing its strength. At the same time, the new way of practicing leadership makes it evident that technologies offer useful support in enlarging and reinforcing relationships within teams; underlying this notion, there is the idea that leadership itself asks for support and points to the search for new opportunities. Regarding competencies, a true e-leader has a clear vision of his/her role in blending traditional and innovative skills, both are necessary to promote team development in a balanced way according to the maturity level of the co-workers in the usage of technological tools. Finally, HR and ICT department support is key to making leadership stronger, both in terms of organizational orientation and within the direct and personal dimension.

## Is E-Leadership a Real Perspective? First Conclusion

The aim of this work was to understand if e-leadership was present or not in Italian enterprises. If so, the task was then to determine where it was “located” in the company, how it developed, and how it was supported; if not, the reason for its absence was to be determined. On this basis, we proposed a framework to clarify “a possible way toward e-leadership” by identifying the operational indications for enterprises interested in establishing whether e-leadership is appropriate for their specific strategies and working conditions.

More specifically, we answered the questions introduced earlier on how the introduction and diffusion of new technologies influenced the organizational leadership system, and, further, how it can advance the usage of new technologies within the perspective of co-evolution and mutual influence, as indicated by the UTAUT.

The importance of the topic with respect to the concrete functioning of organizations justifies a specific interest in the subject, the relevance of which is expected to increase in the life of enterprises. Therefore, managerial implications could be developed to support managers, HR departments, and ICT departments involved in this transformation. We were also interested in considering the implications at the theoretical level that can contribute to the debate on this issue, which continues to attract the interest of scholars in the managerial field.

The general question at the basis of this paper—that is, can e-leadership be considered a real concern and practice in enterprises, and if so, how it is configured—requires an articulated answer.

The first piece of evidence, based on the experiences of the enterprises involved in the analysis, shows that e-leadership cannot be “not present”; its absence seems destined to disappear. From the first to the second phase, the number of companies inserted in the group labeled as “e-leadership not yet present” was reduced. Therefore, it seems evident that this was due to the increase in the pervasiveness of technologies as well as to their diffusion in working life, so that they have necessarily invaded the space of the relationships, which is exactly where leadership is practiced.

Even where e-leadership is not clearly identified and fostered, it insinuates itself, dragged along by the different styles suggested by easy and interactive technology, and is slowly but unavoidably introduced by workers who individually use them, or by an organizational orientation pushing for digital change. Then, it begins to be practiced and is later formalized. In those companies where e-leadership was considered positively and was supported to the extent to which it became diffusely used, e-leadership emerged as a strong reinforcement of the new way to work in organizations, embedding and steering technological change.

In these conditions, it seems that the leader becomes another “figure” solely because of the different context he/she works in, as Hüsing et al. (2013, 2015) have explicitly suggested. This leader’s profile evolves according to the different contexts in which she/he works and to the respective degrees of alignment with the evolution. On the one hand, the social and relational dimension of the organizations (including leadership) are changing by adapting to technologies, and, on the other hand, the social processes (including, of course, leadership) are facilitating confidence with technology.

According to the descriptions we obtained during our interviews, the main features of the emerging e-leadership can be summarized in terms of the following five points. In other words, e-leadership is exemplified by:

• the comprehension of the opportunities offered by ICTs (and not a deep technical expertise) as the basis of an e-leader’s professionalism, so that the UTAUT variable of social influence is reinforced;
• the aptitude toward communication and interpersonal relationships remaining essential, thereby confirming what Van Wart et al. (2019) have suggested: both these dimensions have to be managed in more difficult conditions to avoid the risk of ineffectiveness in comparison with their expression in traditional leadership;
• the orientation toward change, which allows the e-leader to promote a different culture that is accepted easily by digital natives but with more difficulty by the other generations of workers, to whom an innovative and *ad hoc* approach must be proffered;
• the capability to take on risks and to decide quickly, in a fast-moving context;
• the acceptance of a different way of establishing his/her role as a more constitutive skill; this means that the role of the leader is “conquered on the field” in accordance with the demonstrated ability to manage, and it is always less related to the formal position held in the hierarchical line.

In the ideal scenario, a leader is more of a “facilitator” than a “guide,” more of a manager with a different equilibrium—one who is able to effectively blend resources and behaviors—than simply an “attractive boss.” Similarly, team members are less like disciples and more like collaborators. Accordingly, the role of the leader exhibits a change due to the presence of more interactive technologies, the characteristics of which act so deeply on the nature of leadership itself. However, this change is also highly firm specific, embedded within that cultural background and those organizational choices that could help or hamper it. In this perspective, a specific role is played by the UTAUT facilitating conditions, which successfully summarize the organization dimension in the e-leadership process.

In conclusion, the relevance of e-leadership in the concrete functioning of organizations (and its implications on the theoretical level that have yet to be fully developed, as the persistent interest of scholars proves to be done) have been confirmed.

## Limits of the Research and Future Development

The present study has some limitations. The number of the enterprises which have been involved is the first; their location, essentially the Ligurian area, is the second. If the explorative purpose of the analysis justifies the qualitative approach, which allows for the discernment and understanding of soft nuances and weak differences in the enterprises, the limited number of respondents does not help to construct a complete idea of the situation of e-leadership in companies in general.

Moreover, in the second phase, the participants were not the protagonists of the phenomenon we were analyzing, but they were instead the supporters. Even if they belonged to the two most crucial departments for its development, they were expected to stay in second line with respect to the practice of leadership, and they could only propose their points of view with regard to it.

For future research, a deeper understanding of the phenomenon calls for a survey proposed to managers with responsibilities for teams, focusing on their direct experience and the possible support they receive from the HR and ICT departments. Furthermore, more attention to the steps for a possible evolution toward a “full” e-leadership is needed, in addition to a focus on the connection with progressive technological enrichment (both on the quantitative hand—with more devices—and on qualitative hand—in terms of the flexibility and adaptability of devices and apps), because this would illuminate the mutual relationship between the human and technical sides.

Finally, a more detailed lengthwise study could help in examining the evolution of the phenomenon, confirming its relevance, and defining it more accurately.

This study gave us the chance to understand the emerging complexity of the field, which is increasingly dense and diverse in scope. Despite prior citations claiming a dearth of scholarly attention on the topic of e-leadership, we acknowledge the importance of other scholarly publications, in the excluded set of publications, also paying attention to the topic of leaders’ interaction with followers via ICTs. Because of this, we suggest a future research focused on reviewing different constructs which are similar to the one of e-leadership and which are mostly used as synonymous (e.g., digital leadership).

By way of a conclusion, the new circumstances we are experiencing during the COVID-19 pandemic represent a more significant and drastic change in working situations than we could have previously imagined, from which it may be difficult to emerge as if nothing has happened. We are therefore committed to continue this work on e-leadership. It has indeed become an inevitable dimension in working relationships during smart work intensification, even if at an unconscious level by those supervisors who, in this phase, have had to bring out the best in their co-workers by learning and experimenting with e-leadership practices and discovering which of these approaches and organizational conditions make it most effective.

## Data Availability Statement

The raw data supporting the conclusions of this article will be made available by the authors, without undue reservation.

## Ethics Statement

Ethical review and approval was not required for the study on human participants in accordance with the local legislation and institutional requirements. Written informed consent for participation was not required for this study in accordance with the national legislation and the institutional requirements.

## Author Contributions

Although both the authors share the final responsibility for the contents of the manuscript, sections: “The Evolution of Leadership and Its Relationship With Technology”, “Virtual Teams and E-leadership”, “Is E-leadership a Real Perspective? First Conclusion”, and “Main Findings” were written by TT. “Definition of E-leadership” and “Limits of the Research and Future Development” were written by DS. Both the authors co-wrote the sections: “Introduction”, “Empirical Research”, and “Some Answer to Our Questions”. Both authors were also involved in interpreting the data and drafting/critically revising the manuscript.

## Conflict of Interest

The authors declare that the research was conducted in the absence of any commercial or financial relationships that could be construed as a potential conflict of interest.
